# Retraction: MicroRNA-145 down-regulates mucin 5AC to alleviate airway remodeling and targets EGFR to inhibit cytokine expression

**DOI:** 10.18632/oncotarget.28689

**Published:** 2025-02-05

**Authors:** Zhe Cheng, Ling-Ling Dai, Xi Wang, Liu-Qun Jia, Xiao-Gang Jing, Peng-Fei Li, Meng Liu, Huan Wang, Lin An

**Affiliations:** ^1^Department of Respiratory and Critical Care Medicine, Institute of Clinical Medicine, The First Affiliated Hospital of Zhengzhou University, Zhengzhou 450052, P.R. China

**This article has been retracted:** Oncotarget has completed its investigation of this paper, initiated after the request of corresponding author Zhe Cheng to retract this article. Upon investigation, several instances of image duplication were found. In particular, Figure 4, contains images also found in Figure 4 (HE staining), Figure 5 (Masson staining) and Figure 6 (PAS staining) of an earlier paper [1]. Figure 4, HE staining panel and Masson staining panel, also contains images found in Figure 3A and 3C of a paper published concurrently [2] and in Figure 4 of [3]. The authors also reused some of the HE staining and PAS staining images in later published paper [4], which was recently retracted. As a result, the Editorial decision was made to retract this paper. Oncotarget has reached out multiple times to all authors to confirm this retraction, but received no response.

Original article: Oncotarget. 2017; 8:46312–46325. 46312-46325. https://doi.org/10.18632/oncotarget.17933
